# Coagulation testing: Comparison of portable (CoaguChek^®^ XS) and automated coagulation analyzer in healthy cats

**DOI:** 10.14202/vetworld.2020.2541-2545

**Published:** 2020-11-27

**Authors:** Sireeporn Tonthong, Jetsada Rungpupradit

**Affiliations:** Department of Small Domestic Animal and Radiology, Faculty of Veterinary Medicine, Mahanakorn University of Technology, Bangkok, Thailand

**Keywords:** cats, CoaguChek^®^ XS, coagulation testing, point-of-care testing, prothrombin time

## Abstract

**Background and Aim:**

The CoaguChek^®^ XS (CCX) is a portable coagulation analyzer that is widely used to monitor prothrombin time (PT) in human patients taking oral anticoagulants. It can also be reliably used for screening dogs when PT is in the normal range. Efficacy of the portable CCX coagulation analyzer was evaluated for testing PT in healthy cats and the normal range was established.

**Materials and Methods:**

Blood samples of 82 cats were collected from the jugular vein and PT was measured using both the CCX and an automated coagulation analyzer (ACA). Spearman’s correlation was used to measure the strength and direction of association between the two analyzers, while limits of agreement were assessed utilizing Bland-Altman analysis.

**Results:**

Range of PT using the CCX was 10.1-14.1 s. Correlation between the two analyzers was moderate but significant (r=0.3465, p=0.0014). Mean difference between CCX-PT and ACA-PT was 1.624 s and standard deviation was 0.890 with 95.1% of the samples falling within the limits of agreement.

**Conclusion:**

The CCX is a portable, easy to use coagulation analyzer that requires a small volume of blood and gives results within 1 min. Results showed moderate correlation and good agreement with a standard automated laboratory analyzer. The CCX can be used for screening coagulation testing when PT is in the normal range for cats. However, testing accuracy of the CCX in abnormal PT cats should be further investigated before diagnostic coagulopathy applications.

## Introduction

Coagulopathies in cats can occur from inherited congenital coagulation factor deficiencies, rodenticide toxicity, hepatic failure, and disseminated intravascular coagulation [1-7]. Coagulation profile testing is necessary to evaluate these deficits [8]. Testing before an operation is recommended to reduce the risk of uncontrolled bleeding [9]. Automated coagulation analyzers (ACAs) are the standard laboratory method for measuring coagulation profiles. In an ACA, a mixture of thromboplastin and calcium is added to citrated plasma and time taken until clot formation is recorded. When the clot is formed, changing turbidity by solution effects alters the light intensity and this can be detected by the scattered light detection method. The ACA converts light intensity to an electrical signal and reports the result as seconds (s) or international normalized ratio (INR) [10,11]. The INR is typically used in human medicine but not in veterinary practice [12]. One disadvantage of the ACA method is that the PT result has a long turnaround time [13] and this could be problematic for emergency veterinary surgery where laboratories cannot provide this service immediately [14]. Portable point-of-care (POC) coagulation analyzers are widely available and popular in human medicine for timeous evaluation of coagulation profiles [12,15]. Portable analyzers are easy to use, require a small volume of blood, and provide immediate results [15,16]. Many studies have evaluated the efficacy of POC coagulation analyzers compared to the routine laboratory coagulation method [13,15-19]. The CoaguChek^®^ XS (CCX) is a portable coagulation analyzer that was developed for measuring prothrombin time (PT) in patients taking warfarin to prevent coagulopathy after cardiac surgery [20]. The system comprises an analyzer meter and a test strip (plastic cartridge with iron oxide particles). Each test strip has a test area containing a prothrombin reagent (thromboplastin). When blood is applied, the reagent is dissolved and an electrochemical reaction takes place [12,19,20]. The chemical reaction induces the magnetic field which activates and moves the iron oxide particles. A laser photosensor detects the light reflection that occurs from the movement of iron oxide particles until the clot is formed. The time required for this result is known as the PT [12,19].

Accuracy of PT measurement using the CCX was reported in human [17-19], horse [21], and dog [12]. The previous studies found that the CCX was reliable for screening dogs when the PT was normal [12], while it was potentially specific and quite sensitive for detecting abnormal hemostasis in horses [21]. However, the accuracy of the CCX for PT measurement in cats and normal ranges was not evaluated. Before applying the CCX to measure PT, the efficacy and normal range need to be addressed.

The aim of this study was to evaluate the correlation efficiency of the CCX compared to an ACA for measuring PT and establish the normal range of PT in healthy cats.

## Materials and Methods

### Ethical approval

The study was approved by the Animal Care and Use Committee of Mahanakorn University of Technology, Thailand with Protocol number ACUC-MUT-2020/005.

### Animals

Between July 2018 and December 2019, 82 cats were recruited from cases presented for either routine castration or ovariohysterectomy at the Small Animal Teaching Hospital, Faculty of Veterinary Medicine, Mahanakorn University of Technology, Thailand. A permission form was signed by the cat owners to agree to study participation. Medical history, physical examination, and minimum blood profiles such as complete blood count, alanine aminotransferase, and creatinine were evaluated. The laboratory results indicated that all 82 cats were healthy.

### Sample collection and PT recording

Two milliliters of whole blood were collected from the jugular vein of each cat. One drop of blood was immediately used for PT measurement using the CCX portable coagulation analyzer (Roche Diagnostics, Mannheim, Germany), while the remaining blood was mixed into a 3.2% sodium citrate tube and then centrifuged at 3000 g for 15 min. The plasma was harvested and stored at −20°C before testing using an ACA (Sysmex^®^ CS-2500, Siemens Healthcare Diagnostic Products GmbH, Marburg, Germany) within 24 h after collection. The PT values measured by the ACA (ACA-PT) and by the CCX (CCX-PT) were recorded in seconds.

### Statistical analysis

Distribution of continuous data was assessed by the Shapiro–Wilk test and normal ranges (lowest to highest value) of PT from both methods were determined. Correlation of both methods was assessed by Spearman’s correlation test with agreement evaluated using Bland-Altman analysis [22]. Statistical analyses were performed using a commercial software package (NCSS, LLC, Kaysville, Utah, USA) and p*<*0.05 was considered as statistically significant.

## Results

Various breeds were identified in the sample of 49 male and 33 female cats. Ages ranged from 0.5 to 13 years old. The normal range of ACA-PT was 9-12.9 s and the normal range of CCX-PT was 10.1-14.1 s (Table-1). PT results using the portable analyzer were generally higher than the automated analyzer (76/82 samples), except for 3/82 samples that were less than the automated analyzer, while 3/82 samples were equal to the automated analyzer. Coefficients of variation of ACA-PT and CCX-PT were 7.2% and 6.4%, respectively.

**Table-1:** Prothrombin time values measured using an automated coagulation analyzer (Sysmex^®^ CS-2500 System, Siemens Healthcare Diagnostic Products GmbH, Marburg, Germany) and the portable CoaguChek^®^ XS (Roche Diagnostics, Mannheim, Germany).

Analyzer	No of sample	Range (s)
Automated	82	9-12.9
Portable	82	10.1-14.1

**Figure-1:**
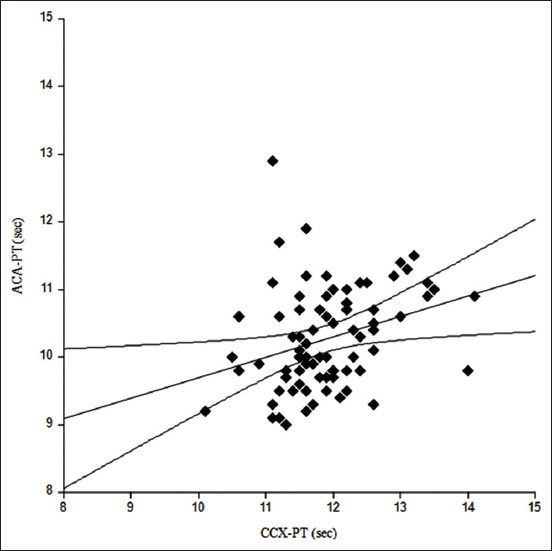
Comparison of prothrombin time (PT) in 82 healthy cats measured using an automated coagulation analyzer and the portable CoaguChek^®^ XS (CCX). Spearman’s correlation test showed that the CCX-PT results had a moderate but significant correlation with the automated coagulation analyzer-PT results in cats (r=0.3465, p=0.0014).

**Figure-2:**
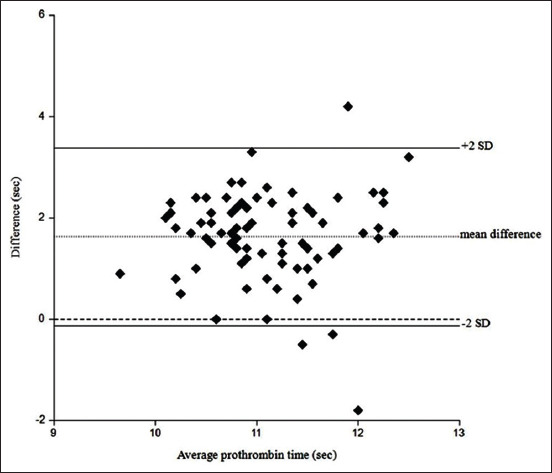
Bland-Altman plot to assess the agreement between CoaguChek^®^ XS-prothrombin time (CCX-PT) and automated coagulation analyzer (ACA)-PT in 82 healthy cats. The X-axis shows the mean (CCX-PT+ACA-PT/2) values in close agreement, representing the accuracy of the results, while the Y-axis shows the difference (CCX-PT - ACA-PT) in values at close to zero with 95.1% of the samples falling within the limits of agreement (1.624 s±2SD).

The Shapiro–Wilk test showed that both ACA-PT and CCX-PT did not have normal distributions (p=0.0177 and 0.0423, respectively). Spearman’s correlation test gave the correlation coefficient (r) and p-value as 0.3465 and 0.0014, respectively (Figure-1). Comparison of PT between both methods was assessed using the Bland-Altman analysis. The mean difference between PT using portable and ACAs was 1.624 s and the standard deviation (SD) was 0.890, while limits of agreement (mean difference±2SD) ranged from −0.12 to 3.37 s. The Bland-Altman plot indicated that 95.1% (78/82) of the samples fell within the limits of agreement (Figure-2).

## Discussion

Efficacy of the CCX compared to the ACA was evaluated for measuring PT in healthy cats. Accuracy was also measured by statistical agreement.

A large number of samples was sufficient to determine an appropriate normal range of ACA-PT and CCX-PT in cats. The results showed the normal range of ACA-PT as 9-12.9 s. Engelen *et al*. [23] determined PT using a STACompact^®^ ACA (Stago Germany, Düsseldorf, Germany) as 10.1-12.8 s, while Stokol *et al*. [24] reported 16-24 s using an STA Compact^®^ (Diagnostica Stago, Parsippany, USA) in healthy cats. Differences in the PT result from various automated analyzers might be influenced by the use of different reagents, especially thromboplastin [25-28]. Therefore, each laboratory should establish its own normal range of PT for a particular test [25].

The normal range of CCX-PT in this study was 10.1-14.1 s as the first report that measured PT using the portable CCX in cats. By contrast, the normal range of CCX-PT in dogs was reported by Kelmer *et al*. [12] as 9.6-11.5 s.

Our study results determined that most samples recorded CCX-PT higher than ACA-PT. Similarly, Newbould and Norman [29] reported that PT using CCX (Roche Diagnostics, Indianapolis, IN) was higher than the standard laboratory result in dogs, while another study in dogs that used a different type of portable coagulation analyzer showed different results, with the POC-PT giving significantly shorter times than results obtained in the clinical laboratory [28]. The difference between CCX-PT and ACA-PT results may be due to several reasons, including species, venipuncture technique, sample handling, different types of portable coagulation analyzer, and methods of endpoint determination [12,28,30]. Consequently, results from the same sample may differ between laboratories.

For non-normal distribution data in this study, a correlation between PT obtained from both methods was assessed by Spearman’s correlation test. The correlation coefficient was determined by the degree of relationship as moderate but significant (p≤0.01), according to Akoglu [31]. Our results were consistent with the previous studies that showed a moderate correlation between portable and ACAs in dogs for the CCX (Roche Diagnostics, Mannheim, Germany) versus ACL 200 (Instrumentation Laboratory, Milano, Italy) (r=0.35) [12], while CCX (Roche Diagnostics, Indianapolis, IN) versus a laboratory model (New Zealand Veterinary Pathology, Palmerston North, NZ) showed high value of coefficient of determination of linear regression (r^2^=0.99) [29].

The Bland-Altman analysis and plot were used to assess the comparability between methods by studying the mean difference and constructing limits of agreement [22]. The previous studies also used Bland-Altman analysis to evaluate the ability of the POC coagulation analyzer for PT measurement compared with standard laboratory methods in dogs. Kelmer *et al*. [12] reported that PT obtained from the CCX (Roche Diagnostics, Mannheim, Germany) provided good agreement with an ACA, while Newbould and Norman [29] reported that PT using CCX (Roche Diagnostics, Indianapolis, IN) had an excellent agreement. In this study, mean difference was 1.624 s and SD was 0.890, with limits of agreement (mean difference±2SD) from −0.12 to 3.37 s. The Bland-Altman plot showed that 95.1% of the samples fell within the limits of agreement. This conformed to statistical recommendations [22] and indicated that PT obtained from the CCX had good agreement with PT obtained from the ACA.

When considering the clinical necessity and benefits of the device, our results suggest that CCX-PT would provide an acceptable and immediate estimation of laboratory PT in healthy cats [22]. Similar to the previous studies, the CCX can be used reliably for screening dogs when the PT is normal [12]. It is useful to rule out coagulation disorder for critical decision making or hemostasis profile testing before an operation. This device is useful for animal clinics in remote areas that are not able to access a veterinary diagnostic laboratory within the required time. The short storage time for the coagulation test and the small sample required for the use of the CCX-PT are also significant advantages compared to the 2 mL of blood required for laboratory testing. The sample required for the CCX is much smaller (8 μL) and can be easier to obtain in cats. In addition, the normal range of PT using CCX in cats was established from this study and will allow use in clinical practice. According to the instruction manual [20], limitations of the CCX are that the device provides an accurate result for hematocrit ranges between 25% and 55%, while the device is not designed to measure results lower than 9.6 s. Further, a high concentration of blood bilirubin, triglycerides, heparin, and severe hemolysis can interfere with the result.

The limitation of this study was that a prolonged PT group of cats was not included. Therefore, the sensitivity and specificity of CCX were not proven. Nevertheless, the previous studies reported sensitivity and specificity of this device as 92% and 56%, respectively, in dogs [12] and 50% and 74%, respectively, in horses [21]. Thus, we do not recommend using CCX for diagnosing coagulation abnormalities in cats. In addition, a recent study that assessed the accuracy of CCX in dogs recommended further investigation of coagulopathy using an automated standard analyzer when CCX-PT was prolonged [12]. Furthermore, this study did not prove that anemia and thrombocytopenia can result in hypocoagulability and lead to false-positive results for POC analyzers in cats, similar to a previous study in canines [12]. Future investigation and clarification of the effect of anemia and thrombocytopenia on CCX-PT results from CCX are necessary.

## Conclusion

The CCX is easy to use, portable coagulation analyzer that requires a small volume of blood and produces immediate results. It can be used to evaluate PT in healthy cats in clinical practice. The results obtained concurred with those measured by a standard automated analyzer. However, for prolonged CCX-PT investigation of coagulopathy, the use of an automated standard analyzer is recommended. Further investigations are required to determine the effects of anemia and thrombocytopenia on the accuracy of CCX-PT results and monitoring the coagulopathy in cats is necessary for more precise diagnoses.

## Authors’ Contributions

Both authors designed the study. ST collected samples and performed the statistical analysis. Both authors drafted and revised the manuscript. JR approved the final manuscript.
